# Supplementary material for: A Missense Variant in PLP2 in Holstein Cattle with X-Linked Congenital Mast Cell Tumor
Source: Animals (Basel). 2022 Sep 7;12(18):2329. doi: 10.3390/ani12182329 (PMC9494957; doi:10.3390/ani12182329)
Supplement: Supplementary file 1 [file animals-12-02329-s001.zip › animals-1832241-supplementary.pdf]

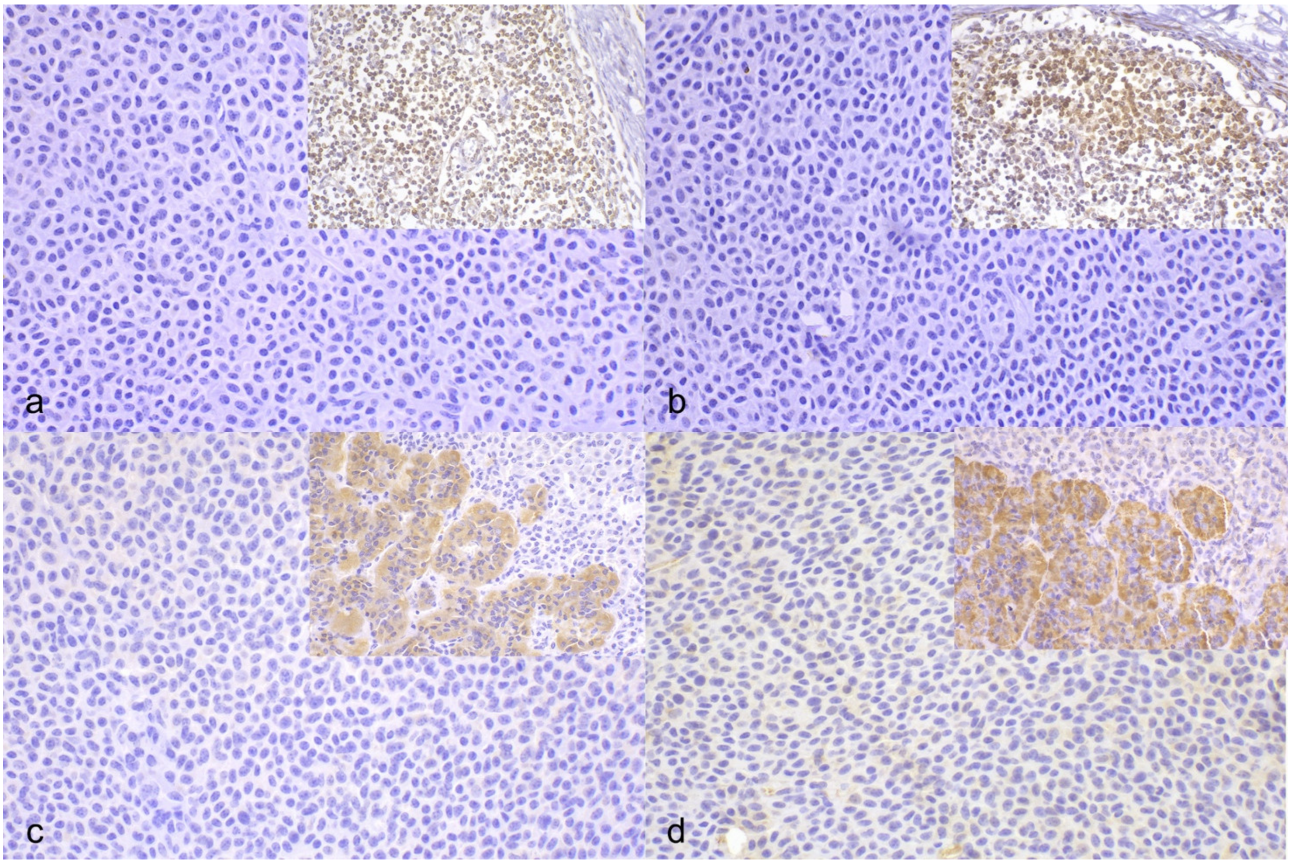

Figure S1. (a) Negative expression of CD3 in the neoplastic cells, 400x; inset CD3 positive control, lymph node. **(b)** Negative expression of CD79 in the neoplastic cells, 400x; inset CD79 positive control, lymph node. **(c)** Negative expression of synaptophysin in the neoplastic cells, 400x; synaptophysin positive control, adrenal gland. **(d)** Negative expression of chromogranin in the neoplastic cells, 400x; chromogranin positive control, adrenal gland.
